# The Great Porn Experiment V2.0: Sexual Arousal Reduces the Salience of Familiar Women When Heterosexual Men Judge Their Attractiveness

**DOI:** 10.1007/s10508-022-02317-4

**Published:** 2022-07-05

**Authors:** Jordan Sculley, Christopher D. Watkins

**Affiliations:** Division of Psychology and Forensic Sciences, Abertay University, Bell Street, Dundee, DD11HG UK

**Keywords:** Internet pornography, Sexual arousal, Face perception, Coolidge effect

## Abstract

Pornography has become widely accessible in recent years due to its integration with the Internet, generating social scientific and moralistic debate on potential “media effects,” given correlations between consumption and various sexual traits and behaviors. One popular public debate (Wilson, 2012) claimed that exposure to Internet pornography has addictive qualities that could impact men’s sexual relationships, underpinned by the “Coolidge effect,” where males are sexually motivated by the presence of novel mates. As claims about Internet and sexual addictions are scientifically controversial, we provide a direct experimental test of his proposal. Adapting a paradigm used to examine “Coolidge-like” effects in men, we examined the extent to which exposure to images of pornographic actresses altered men’s attractiveness ratings of (1) familiar faces/bodies on second viewing and (2) familiar versus novel women’s faces/bodies. Independent of slideshow content (pornographic versus clothed versions of same actress), heterosexual men were less attracted to familiar bodies, and homosexual men were less attracted to familiar women (faces and bodies), suggesting that mere visual exposure to attractive women moderated men’s preferences. However, consistent with one of our preregistered predictions, heterosexual but not homosexual men’s preferences for familiar versus novel women were moderated by slideshow content such that familiar women were less salient on the attractiveness dimension compared to novel women when sexual arousal was greater (pornographic versus clothed slideshows). In sum, our findings demonstrate that visual exposure/sexual arousal moderates attractiveness perceptions, albeit that much greater nuance is required considering earlier claims.

## Introduction

### The Internet, Pornography Consumption, and Human Sexuality

In recent history, pornography consumption has increased in developed nations, as the Internet has afforded easy access to cheap content that has increased in number and diversified thematically (reviewed in Ogas & Gaddam, 2011). Online pornographic content reflects the key themes of group sex, non-heterosexual sex, and paraphilic content across genders and sexual orientations, with separate categories of “female-specific,” for heterosexual men and non-heterosexual women only, and “non-ejaculatory,” for heterosexual women only (Hald & Stulhofer, 2016). Forty-six percent of men and sixteen percent of women report using pornography (e.g., on a weekly basis, Regnerus et al., 2016), which may be driven in part by sex differences in dyadic and solitary sexual desire (Dosch et al., 2016; Hald, 2006). Men primarily consume pornography for masturbation while women primarily consume pornography as part of lovemaking with their partner (Bridges & Morokoff, 2011; Vaillancourt-Morel et al., 2017), with men far more likely to pay for access to pornographic content than women (Ogas & Gaddam, 2011). Men who consume pornography are particularly oriented toward amateur content, while women who consume pornography are particularly oriented toward content involving more than two people (Hald & Stulhofer, 2016). Relative to other exciting content, exposure to erotic content is accompanied by both positive and negative emotions among partnered individuals, such as arousal and a desire to be close to one’s partner, but also negative affect, guilt, and anxiety (Staley & Prause, 2013). Correlates of pornography consumption, in addition to being male, include being younger, politically liberal, non-religious, unpartnered or in an unhappy marriage, having paid or been paid for sex, having committed adultery, and having a relatively good knowledge of computers (Stack et al., 2004). Excessive use of pornography may also be correlated with poor psychosocial functioning and covary with excessive gambling, and use of alcohol, cannabis, and video games (Harper & Hodgins, 2016). In sum, various demographic and behavioral characteristics are correlated with pornography consumption.

Users of pornography may differ in their sexual behaviors and preferences from those who are less inclined to view pornography. For example, pornography consumption is correlated with sexual activity (Stack et al., 2004), number of sexual partners and frequency of casual sex (Braithwaite et al., 2015), and risky sexual behaviors (Prause et al., 2015; Traeen et al., 2015), with this latter outcome moderated by early exposure to pornography and preferences for sexual sensation seeking (Sinkovic et al., 2013), and the extent to which individuals perceive pornography as providing useful sexual information (in a non-monogamous sample of Germans; Wright et al., 2018b). There may be benefits to pornography consumption (Hald & Malamuth, 2008; Miller et al., 2018), such as improving one’s sexual knowledge (Hesse & Pedersen, 2017), and how couples communicate, experiment, and feel comfortable during sex (Kohut et al., 2017). Indeed, qualitative data from large samples suggest that “no negative effects” is the most common response when investigating the effects of pornography on relationships (Kohut et al., 2017). Research focused on mainstream and legal pornography in non-clinical populations does however suggest some negative implications for intimate relationships and sexuality (reviewed in Döring, 2009). For example, although there is no evidence that viewers prefer aggressive pornography or these themes have increased in content over time (Hald & Stulhofer, 2016; Shor & Seida, 2019), consumption may interact with personality traits to predict behaviors such as hostility or sexism toward women, such as when less agreeable men report feeling sexually aroused (Hald & Malamuth, 2015; Hald et al., 2013). Although people tend to attribute negative effects of pornography on other people’s behavior versus their own, pornography consumption may also lead to some sexual or body-related insecurities (reviewed in Döring, 2009). For example, while many sexual concerns are observed independent of pornography consumption, it is related to stronger performance expectations of one’s partner among women, and the likelihood of mental distractions related to men’s sexual performance (Goldsmith et al., 2017), although there is little consistent evidence for negative effects of pornography on heterosexual men’s sexual functioning (Landripet & Stulhofer, 2015). Moreover, pornography viewing has a curved negative relationship on sexual satisfaction that is stronger for men than women, unpartnered versus partnered individuals, and religious versus non-religious individuals (Wright et al., 2018a). Large-scale social survey data from representative samples suggest that declines in sexual frequency over time and between younger versus older generations are not accounted for by pornography consumption, however, where general declines in sexual activity are actually greater among those who abstain from pornography (Twenge et al., 2017). Independent of sexual satisfaction, pornography consumption has a longitudinal effect on marital quality among a representative sample of American couples (Perry, 2017) which is negative for men and positive for women. These findings further highlight the importance of considering the individual, context, and the specific predictor and outcome variables under study, alongside other important moderators such as sexual arousal (Brand et al., 2011; Laier et al., 2013) and qualitative factors (i.e., in a therapeutic context; Gola et al., 2016). Collectively, pornography exposure is related to aspects of sexual relationship functioning, even if the relationship is more complex than a simple stimulus–response model, as implied by “media effects theories” (see, e.g., Ferguson et al., 2017 for a meta-analytic review and discussion of non-pornographic sexual media and adolescent behavior).

### Could Internet Pornography Generate “Coolidge-like” Effects in Humans?

While there are equivocal findings to date on the impact of pornography on sexual behavior and intimacy, one biological phenomenon provides an avenue to examine its potential effects via an experimental design. In 2012, a popular TEDx talk from a self-proclaimed former science teacher (“The great porn experiment,” Wilson, 2012), argued that pornography consumption could have potentially addictive effects for male viewers. Wilson claimed that fast exposure to many different sexually active and receptive females activates reward related areas of the brain and potentially impacts their wider relationships with an individual female partner (e.g., in a more committed context). At the time of writing, his talk had gained over 14 million views on YouTube. Part of his talk rested on the premise that the Coolidge effect (Wilson, 1963) is a biological reality that underpins the enjoyment of pornography by men. The Coolidge effect describes a positive effect of novel female mates on male sexual motivation (e.g., Jordan & Brooks, 2010), which would be adaptive if the potential reproductive rate is higher in males than females across many species. Wilson claimed in his talk that the “unending novelty” associated with pornography consumption may interfere with everyday pleasures experienced during real relationships, as the dopaminergic effects of pornography may be addictive for the male viewer and desensitize them to regular sexual activity. There is evidence that even male primates are motivated to view female genitalia (Deaner et al., 2005), novel erotic stimuli induce “Coolidge-like” effects in men measured via erection (Koukounas & Over, 2000), and learning has an effect on arousal and the sexual response more generally (see, e.g., Pfaus et al., 2001 for review). However, research on Internet addictions (e.g., Przybylski et al., 2017) and sexual addiction are controversial in terms of their scientific validity (Ley, 2014). Thus, experimental tests of various claims made by Wilson are warranted.

### The Current Experiment

Here, we attempt to provide the first direct test of Wilson’s proposal to our knowledge. Namely, that exposure to pornography can generate “Coolidge-like” effects in heterosexual men. Attractiveness and/or valence based on physical appearance is important in social outcomes (Maestripieri et al., 2017; Todorov et al., 2015), social perception (Oosterhof & Todorov, 2008; Sutherland et al., 2013), social memory (Maner & Ackerman, 2015), and the motivation to engage with an individual (Hahn & Perrett, 2014). Much work has also examined how these attractiveness judgments differ according to context (see, e.g., Little et al., 2011 for a review). While familiarity is generally attractive (e.g., Lie et al., 2008; Peskin & Newell, 2004), Little et al. (2014) demonstrated “Coolidge-like” effects in face perception, such that men were more attracted to novelty than women, as indexed by changes in the attractiveness ratings of faces on second viewing. We adapted this paradigm, following our recent work on technology and social perceptions of attractiveness (Sculley et al., 2021), to test whether exposure to pornographic images influences the attractiveness of novel versus familiar bodies and faces. Here, we examined whether our predictions were observed when measuring changes in preferences for the same bodies/faces from baseline (sensu Little et al., 2014), and when comparing the perceived attractiveness of our familiar/test image set to a novel/distractor set of bodies/faces (i.e., familiar versus novel identities).

We preregistered the following hypotheses (Introduction and method section at: https://osf.io/3yr7k/). Based on research on the Coolidge effect in males (e.g., Jordan & Brooks, 2010), “Coolidge-like effects” in men (Little et al., 2014), and the purported effects of Internet pornography on men’s attraction to novel females (Wilson, 2012), we predicted that priming men with images of pornographic content would directly reduce their preferences for familiar bodies on second viewing (i.e., a stronger preference for novelty), compared to our control condition where men view the same actress clothed (Hypothesis 1a). Similarly, when coding data to measure attraction to our novel versus familiar image sets after priming, we predicted the same effect (i.e., stronger preference for novel versus familiar women after exposure to pornography, Hypothesis 1b). Given that the ability to view attractive, nude bodies is a strong motive for viewing pornography, we examined whether the predicted effects were stronger for subsequent attractiveness judgments of bodies versus faces (Hypothesis 2a). Finally, as the hallmark of hardcore heterosexual Internet pornography, compared to sex depicted within film more generally, is arguably the ability to view visible penile–vaginal intercourse, we examined whether our predictions were moderated by pornographic content, such that a stronger cue to sexual availability (observing penile–vaginal intercourse during partnered sex versus solo female nudity) has a stronger effect on men’s preferences for novelty in female bodies (Hypothesis 2b).

## Experiment 1: Lab Experiment on Heterosexual Men

### Method

#### Participants

A total of 129 heterosexual men took part in our laboratory experiment and were recruited from both on and off campus. While we did not exclude participants according to their age or sexuality (i.e., at the recruitment phase), we targeted a sample of heterosexual males aged 18–35 approximately. The study was advertised as examining responses to Internet images (modern technology and social responses to Internet images) and participants were eligible to enter a prize draw for one of two £15 Amazon vouchers. At the consent phase of the experiment, participants were informed that the study may or may not involve looking at sexually explicit images. At the debrief phase of the experiment, participants had the opportunity to withdraw their data given that the project could be argued to entail deception (which was justified to ensure that our manipulation was reliable). No participants withdrew from the research at this point, although data from six participants could not be used due to technical error (two participants) or the participant not following instructions (four participants), resulting in a final sample size for analysis of 123 heterosexual men (*M*_age_ = 24.36 years, SD = 6.18 years). Sample size was calculated based on 80–90% power to detect a moderate effect in an experiment with three between-subjects conditions (i.e., 41–54 participants per cell; Lakens & Evhers, 2014). All procedures for recruitment and testing were granted ethical approval (Approval code: EMS994).

#### Face and Body Stimuli

Using a procedure adapted from Little et al. (2014), participants rated a set of 10 test photographs (5 female faces, 5 female bodies) on two occasions, rating an additional 10 distractor photographs (5 female faces, 5 female bodies) on the second occasion, with each body image belonging to the same woman as each face image (i.e., 10 Caucasian women used in total). All body images were a subset of images from a publicly available image set (3d.sk) as used in Morrison et al. (2017, https://osf.io/8vzwd/), with accompanying face images used in our prior research (e.g., Watkins et al., 2017). All face and body images were taken under standardized conditions. Face images (600 × 800 pixels) consisted of women posing with a neutral expression, no adornments, and hair tied back from forehead. Body images (600 × 800 pixels) consisted of women posing in a standardized star shape front-on to camera with breasts visible but genitals and face obscured. The female test set (*M*_age_ = 24.4 years, SD = 4.34 years; *M*_Attractiveness_ = 4.36, SD = 0.47, *M*_BMI_ = 19.86 kg/m^2^, SD = 1.50) and female distractor set (*M*_age_ = 24.8 years, SD = 2.39 years; *M*_Attractiveness_ = 4.39, SD = 0.31, *M*_BMI_ = 18.65 kg/m^2^, SD = 1.43) were matched on facial attractiveness as rated in Talamas et al. (2016). We matched the two image sets on facial attractiveness rather than body attractiveness given the greater importance of the former over the latter in attractiveness judgments (e.g., Currie & Little, 2009; Furnham et al., 2001). Of note, attractiveness judgments of faces and bodies are underpinned by similar traits (e.g., adiposity, see, e.g., de Jager et al., 2018 for a review) and this design was optimal to test Hypothesis 2a (differences in responses to the same woman in light of modality), while controlling for attractiveness differences between familiar and novel image sets.

#### Pornographic Images

We pilot-tested a set of 57 images belonging to 19 Caucasian female pornographic actors downloaded from a free adult website (hqbabes.com). According to statistics provided by youporn.com, these female actors (*M*_Current age_ = 26.14 years, SD = 3.21 years; *M*_BMI_ = 18.58 kg/m^2^, SD = 1.41), at the time of 30^th^ January 2019, had a total of 1701 videos viewed collectively over 41 million times (*M*_Actor Views_ = 2.2 million SD = 2.1 million; *M*_Actor Views per video_ = 22,970, SD = 23,799; Median Actor Rank = 134, SD = 738). For each actor, we selected an image of the woman: i) clothed without breasts or genitals visible, ii) nude with genitals visible and no other clothing or adornments, and iii) nude, engaging in visible penile–vaginal intercourse (PVI) with a male actor, with 15 of these images picturing sex in a ventro-ventral position. These images were then pilot-tested on a sample of 91 heterosexual males, with each male rating one of three image sets, with trials presented in a random order (650 × 1000 pixels or 1000 × 650 pixels for PVI images) on surveymonkey.com. Participants in the control condition were reimbursed the equivalent of £5 per hour via prolific.ac (*PVI*: 29 males, *M*_age_ = 31.24 years, SD = 5.06 years. Nude: 30 males, *M*_age_ = 32.37 years, SD = 9.22 years. Clothed/Control: 32 males, *M*_age_ = 24.59 years, SD = 6.06 years).

In these pilot studies, men rated each randomized image on the three dimensions of affect used in the International Affective Picture System (i.e., valence, arousal and dominance; Bradley & Lang, 2007) using a 1–9 scale where low scores indicate high valence, high arousal, and low dominance (feeling controlled) respectively. Based on these pilot data, we selected a subset of 15 images per condition with the same 15 female actors used across all three experimental conditions. Here, the two pornographic slideshows differed from the control slideshow on the three dimensions of affect (all *p* < .045, except dominance for Nude v Clothed/Control, where *p* = .07) but did not differ from each other (all *p* > .19) on the three dimensions of affect (PVI slideshow: *M*_Valence_ = 3.83, SD = 0.31; *M*_Arousal_ = 4.28, SD = 0.26; *M*_Dominance_ = 5.79, SD = 0.29. Nude slideshow: *M*_Valence_ = 3.74, SD = 0.29; *M*_Arousal_ = 4.15, SD = 0.26; *M*_Dominance_ = 5.73, SD = 0.34. Clothed/control slideshow: *M*_Valence_ = 4.34, SD = 0.33; *M*_Arousal_ = 4.63, SD = 0.58; *M*_Dominance_ = 5.51, SD = 0.29).

#### Procedure

The experiment was run via Superlab version 4.5 (Cedrus Corporation, San Pedro, California), with a fixation cross-presented in the center of the screen for 200 ms in between all image trials. First, all participants viewed a one-minute slideshow to stabilize baseline levels of arousal across the sample. In this slideshow, we used 12 low-arousal, high-valence nature images presented in a randomized order for 5000 ms each. After reading instructions, participants then proceeded to the first phase of the experiment (pre-priming attractiveness rating task). Participants were presented with 10 randomized trials consisting of five faces and five bodies, with five female models used in total. On each trial, participants were asked to indicate their preference for the face/body on a 1 to 7 scale (much less/more attractive than average). Immediately after this, participants completed the priming phase of the experiment (picture slideshow), with participants blind to the condition they were allocated to. Participants were randomly allocated to one of three conditions (PVI slideshow, Nude slideshow, Clothed/Control slideshow) using the random number generator on Excel. Participants were simply asked to look closely at the images presented within a one-minute slideshow, with trial order randomized and each trial presented for 4000 ms in the center of the screen (520 × 800 pixels or 800 × 520 pixels in the PVI slideshow condition). Immediately after the priming slideshow, participants were asked to rate the same test set of faces and bodies and an additional distractor set of 5 female faces and 5 female bodies, with attractiveness rated in the same way as the pre-priming phase of the experiment and all trials randomized. After this phase, participants were debriefed.

#### Initial Processing of Data and Analysis Plan

Following Little et al. (2014), we calculated each participant’s change in preference for familiar faces/bodies (i.e., from baseline), by averaging their attractiveness ratings of test faces/test bodies. Averages were calculated separately for ratings of the five female faces and the five female bodies and were also calculated separately for the pre-priming phase of the experiment and the post-priming phase of the experiment (i.e., four separate average values). To test our preregistered hypotheses, each participant’s change in preference was calculated by subtracting the participant’s pre-priming score from their post-priming score. High scores (i.e., greater than zero) indicate a stronger preference for familiarity in faces/bodies. Conversely, low scores (i.e., below zero) indicate a stronger preference for novelty in faces/bodies. Average ratings of distractor faces/bodies in the post-priming phase of the experiment were also compared, to examine relative preferences for novel versus familiar women (faces and bodies separately) during the post-priming phase of the experiment.

Mixed-design ANOVAs were run on our two dependent variables, with follow-up *t*-tests with bias correct and accelerated bootstrapped confidence intervals (1000 samples). Two-tailed *p* values were reported for all analyses, with significance set at the level *p* < .05. Data were analyzed from heterosexual males who completed all trials in both phases of our experiment (pre- and post-priming attractiveness ratings of faces and bodies).

### Results

#### Change in Preference for Familiar/Test Images (i.e., Bodies and Faces, from Baseline, Hypotheses 1a, 2a, 2b)

A within-subjects ANOVA was run on the dependent variable change in preference for test image set, with the within-subjects factor modality (female bodies, female faces) and the between-subjects factor experimental priming condition (penile–vaginal intercourse slideshow, nude slideshow, clothed/control slideshow). This analysis revealed an effect of modality that went in the predicted direction (*F*[1,120] = 3.48; *p* = .064, *n*_p_^2^ = 0.03). No effect of experimental priming condition was observed (*F*[2,120] = 1.08; *p* = .34, *n*_p_^2^ = 0.02) or interaction between experimental priming condition and modality (*F*[2,120] = 0.90; *p* = .41, *n*_p_^2^ = 0.02).

**Fig. 1:**
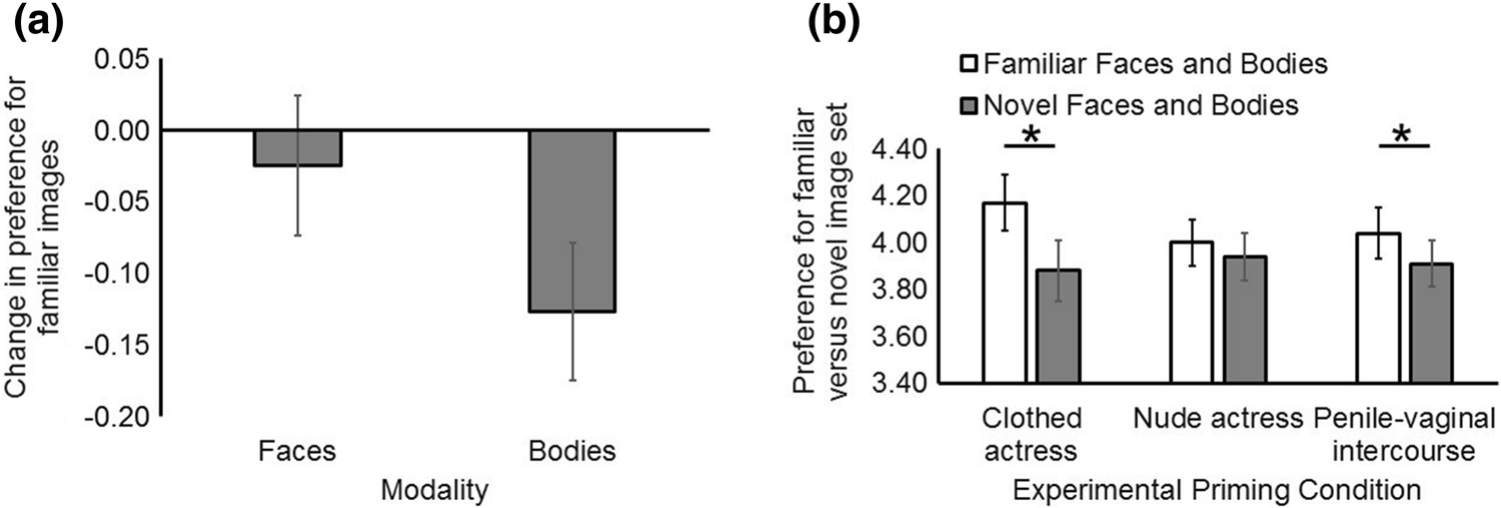
Priming with images of pornographic actresses decreased men’s preference for familiar bodies but not familiar faces (Panel a, *r* = .41). Low arousal was related to a stronger preference for familiar women (i.e., faces and bodies, *r* = .39) while greater arousal reduced the distinction between familiar and novel women on the attractiveness dimension (Panel b). Error bars show ± 1SEM

As our preregistered Hypothesis 2a was directional, we confirmed whether the marginally significant effect of modality went in the same direction as predicted. One-sample *t*-tests examining whether our priming manipulation, in general (i.e., slideshows of pornographic actresses), led to a decrease in preference for bodies but not faces, revealed that priming reduced the attractiveness of familiar bodies (*M* = − 0.13, BCa 95%CI[− 0.22, − 0.03], absolute *t*[122] = 2.64; *p* = .009, *r* = .41) but not familiar faces (*M* = − 0.02, BCa 95%CI[− 0.13, 0.07], absolute *t*[122] = − 0.50; *p* = .62, Fig. 1a).

#### Exploratory Analyses: Change in Preference for Familiar/Test Images (i.e., Bodies and Faces, from Baseline)

We ran further exploratory analyses (one-sample *t*-tests) split by priming condition to rule out potential alternate explanations for our findings, as both of our pornographic slideshows elicited similar levels of arousal during pilot tests, but men may have responded differently to the same woman when she was alone versus partnered (i.e., effects moderated by image content versus sexual arousal, consistent with Hypothesis 2b). This analysis revealed no changes in attractiveness judgments from baseline in any of our slideshows, when men judged faces (all absolute *t* < 0.51, all *p* > .61). Priming with the penile–vaginal intercourse slideshow did not alter body preferences from baseline (*t*[40] = − 0.06, *p* = .95). Priming with the clothed female slideshow did not alter body preferences from baseline (*t*[40] = − 1.81, *p* = .08, *r* = .28). Priming with the nude female slideshow altered body preferences from baseline (*t*[40] = − 2.55, *p* = .015, *r* = .40).

As the penile–vaginal intercourse slideshow did not elicit any change in preference from baseline, and pilot-testing revealed that both pornographic slideshows were equally arousing (but more arousing than our control condition), we repeated our ANOVA on participants who took part in two of our priming slideshows only (nude, clothed/control). Rerunning the ANOVA on these data revealed the predicted effect of modality (*F*[1,80] = 4.64; *p* = .034, *n*_p_^2^ = 0.06) and no other significant effects or interactions (both *F* < 0.37 both *p* > 0.57). This analysis revealed, consistent with the initial preregistered analysis, that body preferences (*t*[81] = − 3.12, *p* = .002, *r* = .35) but not face preferences (*t*[81] = − 0.59, *p* = .56) decreased from baseline across both priming slideshows.

#### Changes in Response Times to Familiar/Test Images (i.e., Bodies and Faces, from Baseline)

Analyses of response time data both for the original preregistered model (*M*_Change in RT_ = − 1.87 s, SD = 2.22 s) and for this exploratory model (*M*_*Change in* RT_ = − 1.87 s, SD = 2.57 s) revealed no significant effects or interactions (all *F* < 2.50, all *p* > .11).

#### Attractiveness of Familiar versus Novel Women Following Exposure to Pornographic Actors (Hypotheses 1b, 2a, 2b)

Next, we ran a separate mixed-design ANOVA on the dependent variable attractiveness of familiar versus novel women following exposure to pornographic actors, with the within-subjects factor modality (female bodies, female faces) and image set (familiar/test set, novel/distractor set) and the between-subjects factor experimental priming condition (PVI slideshow, nude slideshow, clothed/control slideshow). This analysis revealed a main effect of modality (*F*[1,120] = 10.19; *p* = .002, *n*_p_^2^ = .08), which reflected a stronger attraction to faces (*M* = 4.09, BCa 95%CI[3.96, 4.20]) than bodies (*M* = 3.89, BCa 95%CI[3.78, 4.00], *t*[122] = 3.22; *p* = .002, *r* = .14). An interaction between modality and image set was also observed (*F*[1,120] = 77.09; *p* < .001, *n*_p_^2^ = .39), which reflected greater attraction to the novel (*M* = 4.18, BCa 95%CI[4.03, 4.32]) versus familiar face image set (*M* = 4.00, BCa 95%CI[3.87, 4.14], absolute *t*[122] = 3.00; *p* = .003, *r* = .13) but greater attraction to the familiar (*M* = 4.14, BCa 95%CI[4.01, 4.27]) versus novel body image set (*M* = 3.64, BCa 95%CI[3.51, 3.77], absolute *t*[122] = 11.13; *p* < .001, *r* = .45). Consistent with our predictions (Hypothesis 1b), a main effect of image set (*F*[1,120] = 22.33; *p* < .001, *n*_p_^2^ = 0.16) interacted with experimental priming condition (*F*[2,120] = 4.01; *p* < .021, *n*_p_^2^ = 0.06). No other effects or interactions were significant (all *F* < 0.84 all *p* > .43).

Paired *t*-tests split by experimental priming condition were run to examine the significant interaction between experimental priming condition and image set. This analysis revealed that familiar faces and bodies (*M* = 4.17, BCa 95%CI[3.93, 4.42]) were more attractive than novel faces and bodies (*M* = 3.88, BCa 95%CI[3.61, 4.17]) following our control slideshow (i.e., a low arousal priming condition: absolute *t*[122] = 5.41; *p* < .001, *r* = .39). In the opposite direction to that predicted, familiar faces and bodies (*M* = 4.04, BCa 95%CI[3.84, 4.25]) were also more attractive than novel faces and bodies (*M* = 3.91, BCa 95%CI[3.72, 4.11]) following our penile–vaginal intercourse slideshow (absolute *t*[40] = 2.56; *p* < .014, *r* = .20). There was no difference in preference for familiar faces and bodies (*M* = 4.00, BCa 95%CI[3.79, 4.22]) versus novel faces and bodies (*M* = 3.94, BCa 95%CI[3.72, 4.18]) following our nude priming slideshow (absolute *t*[40] = 0.84; *p* = .41, Fig. 1b).

#### Response Time to Familiar versus Novel Women Following Exposure to Pornographic Actors

Response time data on the same ANOVA model revealed a main effect of modality (*F*[1,120] = 12.77; *p* = .001, *n*_p_^2^ = 0.10) and a main effect of image set (*F*[1,120] = 9.91; *p* = .002, *n*_p_^2^ = 0.08), with no other significant effects or interactions (all *F* < 1.86, all *p* > .16). Here, men were faster to respond to the familiar image set (*M*_RT_ = 3.86 s, SD = 1.76 s) than the novel image set (*M*_RT_ = 4.19 s, SD = 2.08 s, *t*[122] = 3.13; *p* = .002, *r* = .14) and were slower to respond to body images after priming (*M*_RT_ = 4.19 s, SD = 1.96 s) than they were to face images after priming (*M*_RT_ = 3.86 s, SD = 1.86 s, *t*[122] = 3.60; *p* < .001, *r* = .16). Excluding participants with response times three standard deviations above the mean (*N* = 13 participants) did not alter the pattern of results reported here for response times, although the main effect of image set now only approached significance (*F*[1,107] = 3.33; *p* = .071, *n*_p_^2^ = 0.03).

## Experiment 2: Effects of Pornography Exposure on Homosexual Men’s Attractiveness Judgments of Familiar versus Novel Women

In order to make stronger inferences about the extent to which our prior effects could be attributed to sexual arousal among heterosexual men, we recruited an independent sample of homosexual men to take part in our experiment, given that, in contrast to women, male sexual arousal is sex-specific independent of male sexual orientation (Chivers et al., 2004).

### Method

#### Participants

A total of 85 homosexual men took part in our experiment. Due to the difficulty in recruiting a large sample of homosexual men on an efficient timescale on campus (for research on a sensitive topic), men were recruited predominantly from LGBQT + locations in the United States and Scotland known to the researcher, with the permission of the owners of the locations. The experiment was advertised and participants were reimbursed in the same way as the first experiment. Three participants withdrew from the research after granting consent, resulting in a final sample size for analysis of 82 homosexual men (*M*_age_ = 36.06 years, SD = 10.21 years). Sample size was calculated in the same way, with all updates to recruitment and testing granted ethical approval (Approval code: EMS2438).

#### Stimuli and Priming Slideshow Images

Stimuli were identical to the first experiment, except that only two slideshow conditions were used (nude versus clothed versions of the same actress), to avoid confounds where homosexual men could be attracted to the male depicted in the partnered (PVI) pornographic slideshow condition.

#### Procedure, Data Processing, and Analytical Strategy

The experimental procedure, and procedure for processing and analyzing data were identical to the first experiment, except that our participants completed the task in a private back booth seating area, in an ambient environment.

### Results

#### Change in Preference for Familiar/Test Images (i.e., Bodies and Faces, from Baseline)

A within-subjects ANOVA on the dependent variable change in preference for test image set with the within-subjects factor modality (female bodies, female faces) and the between-subjects factor experimental priming condition (Nude slideshow, clothed/control slideshow) revealed no significant effects or interactions (all *F* < 2.59, all *p* > .11). A follow-up one-sample *t*-test to examine whether there was a general change in preferences for familiar faces and bodies from baseline (i.e., against zero, collapsed across modality) revealed that men’s preference for familiarity decreased from baseline at levels greater than chance (*M* = − 0.17, BCa 95%CI[− 0.32, − 0.04], absolute *t*[81] = 2.66; *p* = .009, *r* = .14).

#### Changes in Response Times to Familiar/Test Images

**Fig. 2:**
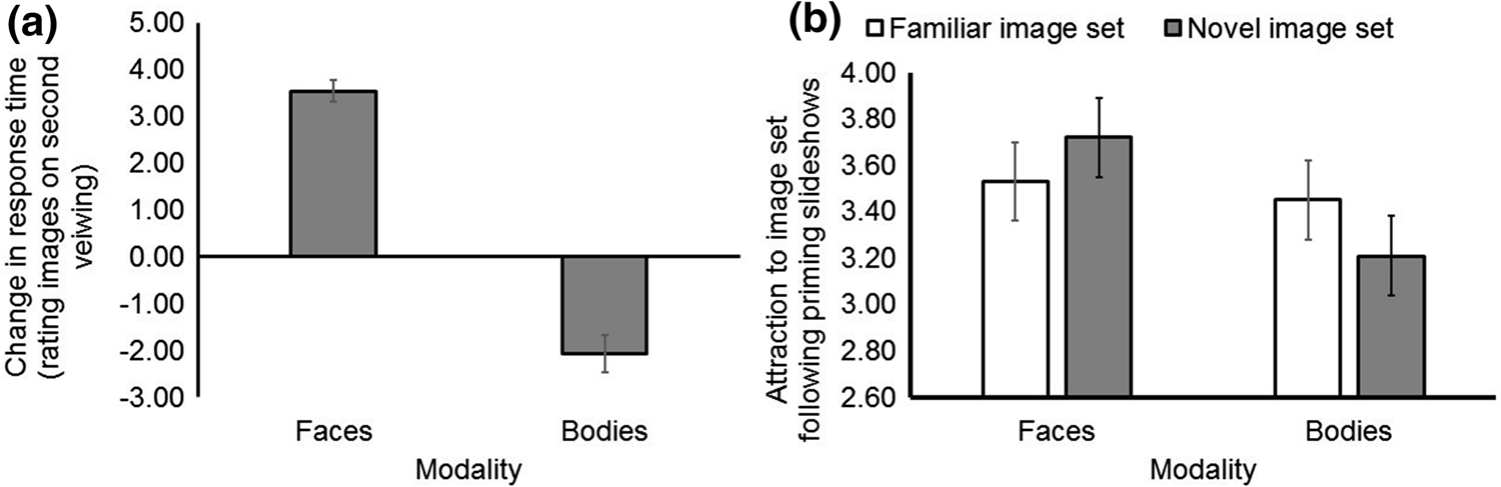
Homosexual men take more time to rate the attractiveness of familiar faces on second viewing and less time to rate the attractiveness of familiar bodies on second viewing (Panel **a**, *r* = .53). Identical to our first experiment (*n*_p_^2^ = .39), homosexual men generally preferred novel faces and familiar bodies (Panel **b**, *n*_p_^2^ = .19). Error bars show ± 1SEM

Running an identical model on changes in response times for this experiment revealed a main effect of modality (*F*[1,80] = 128.27; *p* < .001, *n*_p_^2^ = 0.62) and no other significant effects or interactions (both *F* < 1.03, both *p* > 0.31). This effect of modality reflected an increase in response time when judging the attractiveness of faces on second viewing (*M* = 3.53 s, SD = 2.09 s, *SEM* = 0.23 s) and a decrease in response time when judging the attractiveness of bodies on second viewing (*M* = − 2.07 s, SD = 3.55 s, *SEM* = 0.39 s, absolute *t*[81] = 11.32; *p* < .001, *r* = .53, Fig. 2a). Excluding participants (*N* = 14) above or below three standard deviations from the mean on this measure did not alter this pattern of results (effect size *r* increased to 0.72).

#### Attractiveness of Familiar versus Novel Women Following Exposure to Pornographic Actors

A mixed-design ANOVA was run on the dependent variable attractiveness of familiar versus novel women following exposure to pornographic actors, with the within-subjects factor modality (female bodies, female faces) and image set (familiar/test set, novel/distractor set) and the between-subjects factor experimental priming condition (Nude slideshow, clothed/control slideshow). This analysis revealed a main effect of modality (*F*[1,80] = 7.15; *p* = .009, *n*_p_^2^ = 0.08), which interacted with image set (*F*[1,80] = 18.96; *p* < .001, *n*_p_^2^ = 0.19), reflecting greater attraction to the novel face image set (*M* = 3.72, BCa 95%CI[3.36, 4.08]) than the familiar face image set (*M* = 3.53, BCa 95%CI[3.19, 3.87], absolute *t*[81] = 2.74; *p* = .007, *r* = .15), but greater attraction to the familiar body image set (*M* = 3.45, BCa 95%CI[3.11, 3.83]) than the novel body image set (*M* = 3.21, BCa 95%CI[2.87, 3.62], absolute *t*[81] = 4.17; *p* < .001, *r* = .22, Fig. 2b). No other effects or interactions were significant (all *F* < 2.68, all *p* > .10).

#### Response Time to Familiar versus Novel Women Following Exposure to Pornographic Actors

An identical model on response time data revealed no significant effects or interactions (all *F* < 1.42, all *p* > .23). Excluding participants (*N* = 14), who gave responses more than three standard deviations from the mean response time across trials (7.37 s), did not alter this pattern of results.

### Discussion

#### Summary of Experimental Findings

In our first experiment on heterosexual men, contrary to our preregistered predictions, we did not observe an effect of viewing pornography on men’s preferences for novelty in bodies (i.e., a reduced preference for familiar bodies on second viewing, Hypothesis 1a). Although heterosexual men were less attracted to familiar bodies, but not familiar faces, on second viewing (i.e., in the predicted direction of Hypothesis 2a, see Fig. 1a), this change in preference was independent of the content of our slideshows. Indeed, although exploratory *t*-tests indicated that our sexually arousing slideshow (nude actress) altered body preferences in the predicted direction, while clothed versions of the same actress (low arousal) did not, rerunning our model to compare these two conditions revealed the same general effect on heterosexual men’s preferences. Thus, looking at attractive pornographic actresses who are of high “market demand” (see Pornographic images section of Method) reduced men’s preference for familiar bodies, independent of sexual arousal as confirmed via pilot tests of our slideshows.

When comparing the effects of exposure to pornography on heterosexual men’s preferences for novel versus familiar women (i.e., different image sets), we found partial support for Hypothesis 1b. Although we did not observe any evidence that novel women were perceived as more attractive than familiar women following exposure to pornography, we did find evidence that greater sexual arousal reduced the salience of familiar women relative to novel women on the attractiveness dimension. In other words, familiar women were perceived as relatively less attractive than novel women following exposure to nude or sexually active actresses, compared to when those same actresses were clothed, as evidenced by our significant interaction between image set and experimental slideshow condition (Fig. 1b).

As male sexual arousal is sex-specific regardless of men’s sexual orientation (Chivers et al., 2004), we ran a further experiment on homosexual men to make stronger inferences on whether any effects observed among heterosexual men could be attributed to sexual arousal. Similar to heterosexual men, merely looking at pornographic actresses (independent of image content) reduced their preferences for familiar women, albeit this effect generalized across familiar faces and bodies. When comparing responses to different image sets, we observed an identical interaction to the first experiment, where homosexual men were more attracted to novel versus familiar women’s faces and familiar versus novel women’s bodies (Fig. 2b). However, in contrast to the first experiment, we observed no interaction between image set (familiar versus novel) and experimental priming condition, suggesting that the effects observed in our first experiment could be attributed to sexual arousal (Hypothesis 1b, Fig. 1b). Of note, general time spent looking at women differed according to sexual orientation, following mere exposure to pornographic actresses. Heterosexual men spent longer (330 ms) evaluating women’s bodies versus faces post-priming, while homosexual men spent less time viewing/evaluating familiar bodies, and more time viewing/evaluating familiar faces on second viewing (Fig. 2a, large effect size). This suggests that a different pattern of results emerge for effects of our manipulation on looking/evaluation time versus the attractiveness judgments themselves. Collectively, our data suggest that mere visual exposure to attractive women moderates attractiveness judgments of women regardless of men’s sexual orientation, while sexual arousal via exposure to pornographic content moderates’ men’s perceptions of familiar versus novel women on the attractiveness dimension.

#### Theoretical Implications

There are several implications of our findings. First, although our findings provide some support that exposure to pornography generates “Coolidge-like” effects in heterosexual men (Kokounas & Over, 2000; Little et al., 2014), our data suggest that these effects are much more nuanced than claimed by Wilson in his highly viewed TEDx talk. For example, when examining changes in the perceived attractiveness of the same woman (face or body), our findings could be better accounted for by theories of mere visual exposure on subsequent body perception (e.g., Stephen & Perera, 2014; Sturman et al., 2017) rather than sexual arousal per se, as they were observed in men of both sexual orientations. Indeed, earlier claims are hard to reconcile with our data, as heterosexual men’s preferences for familiar women’s faces did not change following exposure to pornography. This suggests that pornography exposure is unlikely to alter men’s attractiveness judgments of the same woman, and is noteworthy considering the importance of face versus body in social perception (Currie & Little, 2009; Furnham et al., 2001), and as face and body attractiveness are underpinned by similar traits (see de Jager et al., 2018 for a review).

Our second set of analyses, across heterosexual and homosexual men, lends support to the Coolidge effect as a framework for understanding male responses to novel versus familiar women in terms of sexual arousal. Although heterosexual men did not perceive novel women (faces and bodies) as more attractive than familiar women, sexual arousal reduced the perceived distinctiveness of familiar versus novel women on the attractiveness dimension (Fig. 1b). Thus, greater sexual arousal induced by Internet pornography reduces the perceived distinction between familiar versus novel women on the attractiveness dimension. Critically, although mere exposure to pornographic actresses moderated both heterosexual and homosexual men’s preferences for familiar versus novel women in the same manner, preferences for familiar versus novel women were moderated by the content of our slideshows (more versus less arousing) among heterosexual men only. Collectively, our research demonstrates that mere visual exposure to attractive women moderates attractiveness perceptions of bodies more generally, while sexual arousal moderates heterosexual men’s preferences for familiar versus novel women. While the former result suggests that media stimuli in general influences attractiveness perceptions of bodies, pornographic content has specific effects on heterosexual men’s comparative judgments of women, at least in the short term.

#### Limitations and Future Directions

There are limitations to our research. First, our main experiment was limited to adult males in their mid-20s, albeit our randomized slideshow design rules out differential responses to our slideshows that are mere artifacts of demographic characteristics. Nonetheless, it would be useful to examine whether our effects are stronger or weaker according to age (older-age and/or adolescent samples). Considering factors such as level of pornography consumption, sexual disgust, age at first exposure to pornography, and socioeconomic status would also be useful in future. Of note, familiarity effects can be observed after relatively short exposure durations (Montoya et al., 2017), and our data suggest that participants could differentiate two image sets based on familiarity following a brief (one-minute) experimental slideshow manipulation, as their attractiveness judgments of them differed even though the two image sets were equivalent in facial attractiveness. Nonetheless, it would be useful to extend our line of research to examine the extent to which our manipulation influences perceptions of personally familiar individuals. Given that men’s face ratings of the same woman did not change on second viewing, for example, it might be unlikely that the same manipulation would alter perceptions of faces for whom the participant is romantically attached (versus attracted) to. Indeed, “Coolidge-like” effects in men’s judgments of faces appear to be stronger when men are given contexts to evaluate faces related to short-term sexual attraction versus long-term commitment, and men also perceive faces that resemble their romantic partners as more attractive than faces that share less resemblance (Little et al., 2014). Thus, our findings may speak more directly to contexts prior to commitment and the potential (subtle) interplay between men’s sexual experiences online and offline. As affective experiences with faces can be generalized to novel faces that share resemblance to learned faces (Verosky & Todorov, 2010), it is possible that simulated sexual experiences online may influence men’s partner search for specific traits, such as learned traits of positive valence when using online dating sites. Manipulating the exposure duration to faces/bodies and actresses would also be useful, to understand the temporal dimensions of our effects, which may be more substantial in the real world as our experimental manipulation was brief. Manipulating the relative familiarity versus novelty of faces would also be an important means with which to develop this line of research, in contrast to the one- versus two-time exposure used here. This could be manipulated, for example, with an “*x* versus 3 × exposure,” or by manipulating the extent of the morph between a new and old face (Verosky & Todorov, 2010).

Second, our experiments exposed men to images of pornographic actresses. This was done to improve internal validity, where men are exposed to the same actress who is either clothed, fully naked with genitals visible, or naked and engaged in visible penile–vaginal intercourse (i.e., removing confounds related to actress identity, body size, and shape). Exposure to the latter is arguably the hallmark of hardcore heterosexual Internet pornography in contrast to how sex scenes are typically depicted on non-paywalled programs on television. Although future work could examine whether our effects generalize to watching videos and/or more interactive forms of pornography (e.g., via webcam or VR; see Elsey et al., 2019), such a design can induce noise, as dynamic images bring other factors/confounds into play, such as an individual male’s preference for a given theme/style of pornography, the expressiveness or enjoyment (or lack thereof) of different actors, sexual positions used, and so forth. As our tightly controlled experiment exposed men to a relatively reduced range of sexual arousal via picture slideshows, further research such as this would allow us to examine whether our effects are attenuated or accentuated at much lower and higher levels of arousal, even though relatively small changes to slideshow content (one-minute slideshows of the same women clothed versus nude) were sufficient to alter heterosexual but not homosexual men’s preference for familiar versus novel women. Manipulating the attractiveness of the actresses within the stimulus set, and measuring the participant’s own attractiveness, would also be important, to examine whether our effects generalize across a wider range of sexual attractiveness, which may partly be reflected in different styles of pornography (e.g., “amateur” or “girl next door” content; Hald & Stulhofer, 2016).

Finally, the findings from the experiments presented here cannot be generalized to how women interact with pornography. We focused on males in light of prior theory, and due to the practical (and potential ethical) difficulties of running an identically designed experiment on women, as prior work on the sex specificity of sexual arousal (Chivers et al., 2004) means that it would be difficult to conclude from images of dyadic sex whether women’s subsequent preferences are influenced by the actor or actress. Lack of consent from an academic testing platform prohibited us from running an equivalent experiment on women with no experimenter interaction (i.e., maximum privacy). Fewer women than men watch pornography (Albright, 2008; Ogas & Gaddam, 2011), with qualitative research suggesting that women are less motivated to view male genitalia than men are to view female genitalia (Eck, 2003). Indeed, attractive women are in receipt of more prosocial biases toward them than are attractive men (see Maestripieri et al., 2017 for discussion), which may be an important driver in the greater market demand of female versus male pornographic actors. Nonetheless, further research on women’s judgments would be necessary, particularly as reported pornography use is quite high for both men and women in some samples (e.g., 91.5% of men and 60.2% of women report consumption in the past month; Solano et al., 2020). This could be done, for example, by adapting our paradigm to look at women’s responses to literary pornography (see Döring, 2009 for review), or by considering motives to view pornography that are shared by men and women, such as sexual curiosity and self-exploration (Bothe et al., 2021). Pending further independent replication, our findings have practical implications. By integrating these findings with research on training programs related to sex education, researchers and practitioners may make more careful or nuanced conclusions on the effects of this media on behavior, when considering the literature as a whole.

### Conclusion

In sum, our findings suggest that mere exposure to pornographic actresses directly reduces the attractiveness of familiar bodies on second viewing, independent of men’s sexual orientation. Although none of our male samples perceived novel women as more attractive than familiar women following exposure to pornographic images, heterosexual men perceived familiar versus novel women as less distinct on the attractiveness dimension when sexual arousal was relatively high.

## Data Availability

Data and codebook are available via the Open Science Framework (https://osf.io/3yr7k/).
